# Evolution of Cortical Functional Networks in Healthy Infants

**DOI:** 10.3389/fnetp.2022.893826

**Published:** 2022-06-15

**Authors:** Derek K. Hu, Parker W. Goetz, Phuc D. To, Cristal Garner, Amber L. Magers, Clare Skora, Nhi Tran, Tammy Yuen, Shaun A. Hussain, Daniel W. Shrey, Beth A. Lopour

**Affiliations:** ^1^ Department of Biomedical Engineering, University of California, Irvine, Irvine, CA, United States; ^2^ Division of Neurology, Children’s Hospital Orange County, Orange, CA, United States; ^3^ Division of Pediatric Neurology, University of California, Los Angeles, Los Angeles, CA, United States; ^4^ Department of Pediatrics, University of California, Irvine, Irvine, CA, United States

**Keywords:** electroencephalography, functional connectivity, brain mapping, pediatrics, resting-state networks, graph theory, development

## Abstract

During normal childhood development, functional brain networks evolve over time in parallel with changes in neuronal oscillations. Previous studies have demonstrated differences in network topology with age, particularly in neonates and in cohorts spanning from birth to early adulthood. Here, we evaluate the developmental changes in EEG functional connectivity with a specific focus on the first 2 years of life. Functional connectivity networks (FCNs) were calculated from the EEGs of 240 healthy infants aged 0–2 years during wakefulness and sleep using a cross-correlation-based measure and the weighted phase lag index. Topological features were assessed *via* network strength, global clustering coefficient, characteristic path length, and small world measures. We found that cross-correlation FCNs maintained a consistent small-world structure, and the connection strengths increased after the first 3 months of infancy. The strongest connections in these networks were consistently located in the frontal and occipital regions across age groups. In the delta and theta bands, weighted phase lag index networks decreased in strength after the first 3 months in both wakefulness and sleep, and a similar result was found in the alpha and beta bands during wakefulness. However, in the alpha band during sleep, FCNs exhibited a significant increase in strength with age, particularly in the 21–24 months age group. During this period, a majority of the strongest connections in the networks were located in frontocentral regions, and a qualitatively similar distribution was seen in the beta band during sleep for subjects older than 3 months. Graph theory analysis suggested a small world structure for weighted phase lag index networks, but to a lesser degree than those calculated using cross-correlation. In general, graph theory metrics showed little change over time, with no significant differences between age groups for the clustering coefficient (wakefulness and sleep), characteristics path length (sleep), and small world measure (sleep). These results suggest that infant FCNs evolve during the first 2 years with more significant changes to network strength than features of the network structure. This study quantifies normal brain networks during infant development and can serve as a baseline for future investigations in health and neurological disease.

## 1 Introduction

The development of the infant brain (0–2 years old) is characterized by the evolution of neuronal oscillations in various frequency bands, which can be measured non-invasively using electroencephalography (EEG). Such cortical rhythms can be mapped as functional connectivity networks (FCNs) based on the statistical relationships between these neuronal oscillations across spatially distinct regions. Prior studies have reported strong EEG functional connections predominantly in the frontal and parieto-occipital regions in newborn infants (Omidvarnia et al., 2014; Tóth et al., 2017), with a subsequent shift in network topology from a randomized structure towards a more efficient and organized network during infancy (Xie et al., 2019) and childhood (Boersma et al., 2011). These FCNs also have properties that are specific to certain EEG frequency bands. Newborn infants exhibit clustered, fronto-parietal connections in the theta and alpha bands (Tóth et al., 2017). From infancy to age 18, healthy subjects show an increase in broadband connectivity strength, decrease in high gamma connectivity strength, decreases in delta and theta band clustering, and increase in gamma band clustering (Chu et al., 2014). Such frequency band-specific FCNs can indicate different neural mechanisms, as delta and theta networks integrate long-range neuronal assemblies, while gamma and higher frequencies reflect more localized networks (Fries, 2005; Buzsáki and Watson, 2012; Fries, 2015; Bastos and Schoffelen, 2016).

While studies have examined EEG FCNs around the time of birth (Omidvarnia et al., 2014; Tokariev et al., 2016; Tóth et al., 2017), within a span of several months during infancy (Xie et al., 2019), or during early childhood (Boersma et al., 2011; Bathelt et al., 2013), there has yet to be a study that focuses exclusively on the evolution of connectivity during the first 2 years of life. Chu et al. (2014) analyzed EEG connectivity in this age range, but primarily focused on developmental changes across a wider age range of 0–18 years. Moreover, no consensus has been reached on the typical developmental changes in FCNs. Small-world topological features have been reported to disappear around 10 months of age (Xie et al., 2019), despite reports of small world networks in older children (Boersma et al., 2011; Bathelt et al., 2013). Graph theory (GT) measures in FCNs such as path length have also produced mixed results, with both decreases (Miskovic et al., 2015) and increases (Boersma et al., 2011) in alpha band path length with age during childhood. Increases in clustering coefficient and path length from childhood to adolescence have been reported (Smit et al., 2012), while other studies found no correlations between subject age and GT measures in childhood (Bathelt et al., 2013). These differing results may be attributed to several factors, including the variance across EEG datasets and the choice of functional connectivity technique, e.g., linear versus nonlinear, bivariate versus multivariate, and phase-based versus amplitude-based measures (Olejarczyk et al., 2017; Siems and Siegel, 2020).

Therefore, the goal of this study was to characterize the changes in functional connectivity networks over the course of normal infant development using two complementary computational methods. We measured EEG FCNs in a large cohort of healthy infants (*n* = 240), ranging from 0 to 24 months old. For each subject, networks were derived separately for wakefulness and sleep in the delta, theta, alpha, beta, and broadband frequency bands. Two complementary methods were used to calculate the FCNs: cross-correlation and weighted phase lag index. Differences between age groups were quantified using connectivity strength and GT measures. By focusing specifically on healthy infants and directly comparing results across various connectivity techniques, this work further elucidates the evolution of FCNs during normal brain ontogeny and serves as a baseline for the study of early life neurological diseases.

## 2 Materials and Methods

### 2.1 Subject Information and Electroencephalography Recordings

Approval for this retrospective observational study was obtained from the Institutional Review Board of the Children’s Hospital of Orange County (CHOC), with the requirement for informed consent waived. A total of 240 subjects aged 0–24 months were retrospectively identified from the clinical record at CHOC, with visits between 1 January 2012 and 1 January 2019. Subjects were included if they had 1) no known neurological disorders, 2) routine EEG studies that were interpreted as normal by a board-certified pediatric epileptologist (DS), 3) no use of neuroactive medications, and 4) no premature birth (gestational age >38 weeks). Subjects were divided into eight age groups (*n* = 30 for each group) in 3-month intervals (e.g., 0–3 months, 3–6 months, etc.) based on the subject’s age at the time of EEG recording. Three subjects in the 0–3-months age group were excluded from the analysis due to excessive artifactual noise across multiple channels. The demographics of the study population are summarized in Table 1.

**TABLE 1:** Participant demographics.

Group	Age in mos. *M* (SD)	Female *n* (%)	Subjects *n*	Wakefulness *n* (%)	Sleep *n* (%)
0–3 m	1.54 (0.94)	12 (40.00)	30	24 (80.00)	22 (73.33)
3–6 m	4.67 (0.93)	16 (53.33)	30	29 (96.67)	26 (86.67)
6–9 m	7.41 (0.83)	19 (63.33)	30	30 (100.00)	29 (96.67)
9–12 m	10.24 (0.74)	19 (63.33)	30	29 (96.67)	24 (80.00)
12–15 m	13.57 (0.87)	17 (56.67)	30	29 (96.67)	23 (76.67)
15–18 m	16.33 (0.97)	15 (50.00)	30	28 (93.33)	27 (90.00)
18–21 m	19.01 (0.73)	17 (56.67)	30	29 (96.67)	22 (73.33)
21–24 m	22.54 (0.92)	16 (53.33)	30	29 (96.67)	24 (80.00)

### 2.2 Electroencephalography Acquisition and Preprocessing

EEG data were recorded with a Nihon Kohden EEG acquisition system, with nineteen scalp electrodes (Fp1, Fp2, F3, F4, C3, C4, P3, P4, O1, O2, F7, F8, T3, T4, T5, T6, Fz, Cz, and Pz) placed according to the international 10–20 system, at a sampling rate of 200 Hz. One subject’s EEG was recorded at 500 Hz and downsampled prior to analysis. All EEG studies lasted from 20 to 70 min and contained a mixture of wakefulness and sleep. Manual EEG sleep staging was performed for all subjects by registered polysomnographic technologists (CG, AM, and NT) in accordance with the American Academy of Sleep Medicine (AASM) guidelines. EEG studies for subjects younger than 3 months were scored as wake, active sleep, or quiet sleep according to standard criteria. For all subjects older than 3 months, EEG epochs were categorized as wake (W), rapid eye movement (REM), non-REM stage 1 (N1), non-REM stage 2 (N2), and non-REM stage 3 (N3) sleep. However, only wake and N2 sleep data were analyzed, as most subjects’ studies contained a sufficient quantity of these stages for the connectivity analysis. N2 sleep was also chosen due to its high inter-scorer reliability during sleep staging and because of the stability of the FCN during this sleep stage in individual subjects over time (Chu-Shore et al., 2012; Rosenberg and Van Hout, 2013).

Time periods in the EEG containing artifact were identified using an automatic extreme value detection algorithm similar to prior studies (Durka et al., 2003; Moretti et al., 2003; Smith et al., 2021). To identify artifacts, the raw data were filtered using a broadband bandpass filter (1.5–40 Hz Butterworth filter), re-referenced to the common average, and normalized in each channel to have zero mean and unit variance. In each channel, artifacts were identified as periods where the absolute value of the voltage exceeded a threshold of 7.5 standard deviations above the mean value, with a buffer of 0.9 s added to the beginning and end of each period. Impedance checks and photic stimulation were visually identified in the EEG and were also marked as artifact. For all subjects, the mean duration of EEG recordings and time in wakefulness and quiet/N2 sleep is provided in Supplementary Table S1, and the percentage of artifactual data in the EEG recordings is given in Supplementary Table S2.

For the connectivity analysis, the raw data were re-referenced to the common average and filters were applied for each connectivity technique as described in Section 2.3. Time periods containing an artifact in at least one channel were then removed from all channels. For each sleep stage, clean EEG segments with no detected artifact were then separated into two-s epochs for FCN analysis. All electronic data were deidentified and analyzed offline using custom MATLAB (Mathworks) scripts.

### 2.3 Functional Connectivity Network Calculations

Two functional connectivity metrics were used in this study: cross-correlation (CC) and weighted phase lag index (wPLI). CC is a linear, time domain measure applied to broadband data, and wPLI is a phase-based measure applied to individual frequency bands that is sensitive to both linear and nonlinear interactions (Vinck et al., 2011; He et al., 2019). For CC, broadband EEG data were analyzed after zero-phase shift digital filtering from 0.5 to 55 Hz; wPLI was analyzed in the delta (2–4 Hz), theta (4.5–7.5 Hz), alpha (8–12.5 Hz), and beta (13–30 Hz) frequency bands. For the FCN calculation of each subject, 120 two-s epochs of clean EEG data were randomly selected within each brain state (wakefulness and quiet/N2 sleep), and CC and wPLI were calculated independently for each brain state. The number of epochs was chosen based on an analysis of the stability of the connectivity technique; if a subject had insufficient clean data for a particular brain state, their data were excluded from analysis. The number of subjects included in the FCN analysis for wakefulness and quiet/N2 sleep is shown in Table 1.

#### 2.3.1 Cross-Correlation

Cross-correlation is a linear measure of connectivity based on the maximal cross-correlation between two EEG channels at non-zero lags (Kramer et al., 2009; Chu-Shore et al., 2012). This technique has been previously applied to both epileptic and healthy infant EEG data (Shrey et al., 2018; Hu et al., 2020; Smith et al., 2021). For each subject and brain state with sufficient data, we began the connectivity analysis by subdividing the 120 two-s epochs into 240 one-s epochs. The choice of epoch length for CC was based on prior work demonstrating the stability of this measurement for as few as ∼100 epochs of one-s duration (Chu-Shore et al., 2012; Shrey et al., 2018; Smith et al., 2021). The connectivity for each one-s epoch was calculated as the maximal absolute value of the cross-correlation with a maximum lag time of ±200 ms. Additional steps accounted for the influence of volume conduction and the autocorrelation of each signal, and permutation resampling was used for significance testing; please see Hu et al. (2020) for details. The CC connectivity for each subject was reported as an adjacency matrix where each element represented the percentage of epochs with significant connectivity values, with values ranging from zero to one for each pair of channels.

#### 2.3.2 Weighted Phase Lag Index

The wPLI is a measure of functional connectivity based on the phase synchronization between channel pairs. For channels x and y , the wPLI in a data segment with n time points is defined as:

$$\mathrm{wPL}I_{xy}=\;\frac{n^{-1}\sum_{t=1}^{n}\left|imag\left(S_{xyt}\right)\right|sgn\left(imag\left(S_{xyt}\right)\right)}{n^{-1}\sum_{t=1}^{n}\left|imag\left(S_{xyt}\right)\right|}$$ (1)

The wPLI measures the average sign of the imaginary component of the cross spectrum $S_{xy}$ and weights the value by the magnitude of the imaginary component to reduce the effect of cross-spectral values near zero and pi. The wPLI was chosen because it is more sensitive to phase synchronization than PLI, while reducing the influence of both noise sources and volume conduction (Vinck et al., 2011).

For each subject, all 120 two-s epochs of clean EEG data were used to calculate the wPLI FCN, as this amount of data ensures the stability of the measure (Haartsen et al., 2020). In each frequency band, the instantaneous phase was obtained *via* the Hilbert transform of the filtered EEG signal for each channel. A connectivity matrix was then calculated for each epoch using Eq. 1 for each channel pair. The significance of each epoch was assessed by generating a null distribution of wPLI values for each pair of EEG channels under the assumption of no temporal relationship between the signals. This was done by calculating the wPLI of two randomly selected epochs for 1,000 iterations. The measured wPLI value for each channel pair in an epoch was considered significant if it exceeded the 95th percentile of the null distribution. Significant connections were assigned a value of one, while non-significant connections were assigned a value of zero. The FCN for each subject was then calculated by averaging the binary matrices over all epochs. Therefore, analogous to the CC measure, each element in the adjacency matrix represents the percentage of epochs with significant connectivity values.

### 2.4 Graph Theory Metrics

Graph theory has been successfully used to analyze networks in a broad range of human neuroscience studies (Sporns, 2018), with applications to data modalities ranging from structural and functional brain measurements (Hallquist and Hillary, 2018; Bellantuono et al., 2021) to genetics (Monaco et al., 2019). Here, differences in FCNs between groups were quantified using four different GT measures: 1) network strength, 2) normalized global clustering coefficient (nGCC), 3) normalized characteristic path length (nCPL), and 4) small-world measure (SW). These measures were found to be relevant in prior studies of neonatal (Omidvarnia et al., 2014; Tóth et al., 2017), infantile (Fan et al., 2011; Gao et al., 2011; Xie et al., 2019), and childhood development (Boersma et al., 2011; Bathelt et al., 2013). All metrics were calculated using functions from the Brain Connectivity Toolbox (Rubinov and Sporns, 2010) and custom MATLAB (Mathworks) scripts.

The network strength was defined as the mean of the strongest ten percent of connections and was calculated for each subject, connectivity technique, and brain state (Garrison et al., 2015; Smith et al., 2021). Topological changes in the network structure were quantified using GCC and CPL. A network with a high GCC indicates the presence of strongly connected electrode triplets, and a low CPL value indicates that the network can efficiently transfer information between nodes (Watts and Strogatz, 1998). To reduce the effects of weak connections, the weighted connectivity matrices were thresholded at an edge density of 0.25. This threshold was chosen by testing edge densities from 0.05 to 0.80 (Supplementary Figures S1–S5). We found that edge densities under 0.1 had high variability and often resulted in unconnected graphs, while edge densities over 0.4 could produce fully connected graphs with nGCC and SW values near one. An edge density of 0.25 was chosen here, as it exhibited low variability between subjects while forming a connected graph. In addition, prior studies found that edge densities of 0.1–0.4 were appropriate for GCC, CPL, and SW measures (Mehraram et al., 2020; Carpels et al., 2021). The thresholded weighted matrix was then normalized by dividing each element by the maximum connectivity value to avoid the influence of network strength on the GT measures (Onnela et al., 2005; Antoniou and Tsompa, 2008; Mehraram et al., 2020; Carpels et al., 2021). This normalized matrix was used to calculate GCC, and it was also converted into a distance matrix to calculate CPL. The GCC and CPL measures were then reported as normalized values relative to 100 surrogate networks generated using an Erdős–Rényi random graph:

$$nGCC=\frac{GCC}{{GCC}_{rand}}$$ (2)

$$nCPL=\frac{CPL}{{CPL}_{rand}}$$ (3)

An nGCC value greater than one suggests that the network is more clustered than a randomized network, while an nCPL greater than one suggests that the network is configured to transfer information less efficiently than a randomized network.

The small-world characteristics of the FCNs were measured as the ratio of the normalized GCC and normalized CPL measures (Humphries and Gurney, 2008):

$$SW=\;\frac{nGCC}{nCPL}$$ (4)

Small-world networks are typically characterized by high values of GCC and low values of CPL. Compared to a randomized network with low clustering and short path length, a small-world FCN should then have an SW > 1.

### 2.5 Statistical Tests

Statistical analyses of the GT measures were conducted using one-way ANOVA tests across the eight age groups. Results were calculated independently for each connectivity method, frequency band, and sleep stage. The Bonferroni method was used to correct for multiple comparisons, accounting for 28 different age-group comparisons. The SW comparisons between CC and wPLI were measured in each age group using a one-tailed Wilcoxon signed-rank test, corrected using the Bonferroni method.

## 3 Results

### 3.1 CC FCNs Increase in Strength in Early Infancy

**FIGURE 1:**
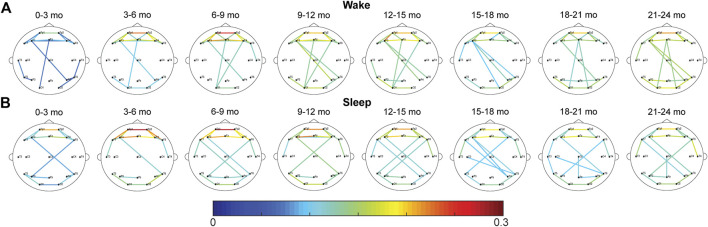
Average CC FCNs for healthy infants during **(A)** wakefulness and **(B)** sleep. The strongest 10% of connections in each age group are shown. The color of each connection represents the connection strength, defined as the percentage of epochs with significant connectivity values.

**FIGURE 2:**
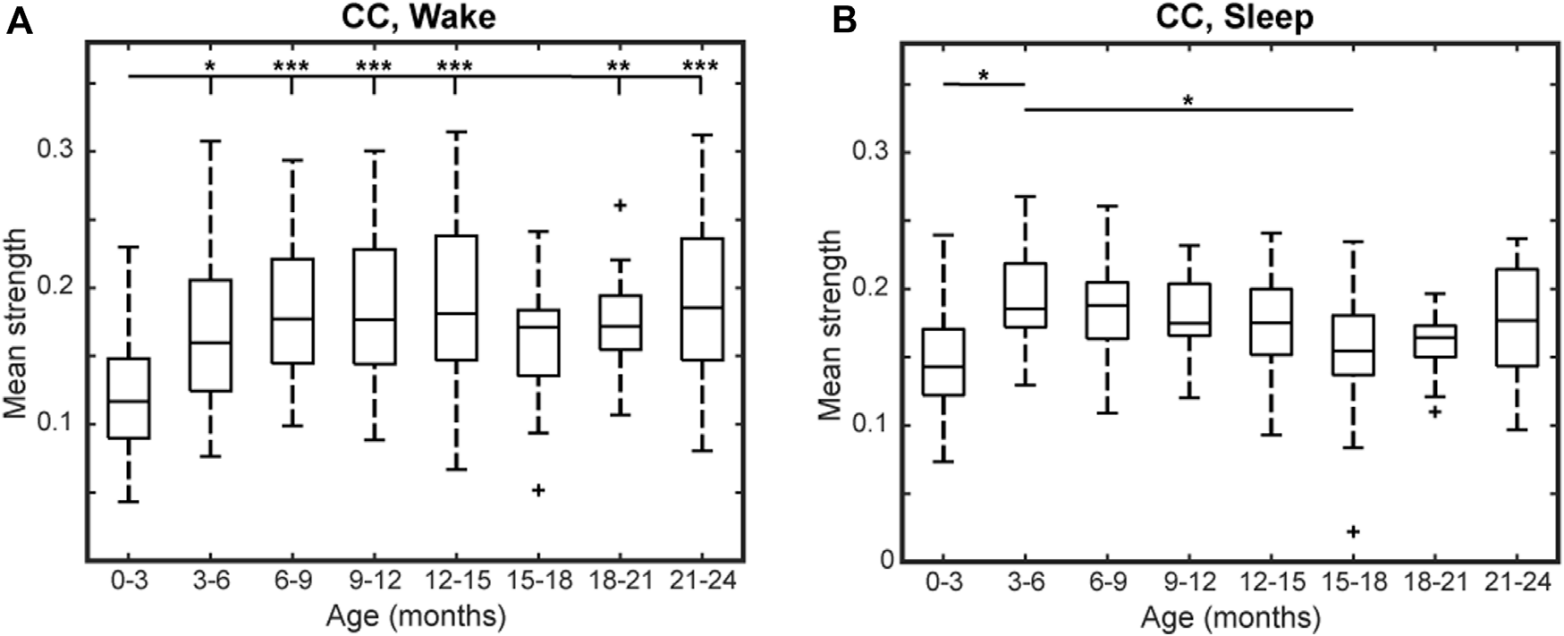
Mean network strength by age for healthy infants using CC connectivity during **(A)** wakefulness and **(B)** sleep. Here, mean network strength is defined as the mean of the 10% highest connectivity values. Significance levels are *p < 0.05, **p < 0.01, and ***p < 0.001, with *p*-values modified using the Bonferroni method.

For the CC FCNs, both frontal connections (between electrodes Fp1, Fp2, Fz, F3, F4, F7, and F8) and occipital connections (between O1, O2, T5, and T6) were frequently among the top 10% strongest connections in the network; this was true across all age groups, during wakefulness and sleep (Figure 1). However, the mean network strength increased after the first 3 months of infancy, as significant differences were found between subjects 0–3 months old and subjects 3–15 and 18–24-months old in wakefulness (*p* < 0.01, Figure 2A) and between 0–3 months old and 3–6-months old in sleep (*p* < 0.05, Figure 2B). For subjects older than 3 months, the only significant difference in network strength was a lower strength in subjects 15–18 months old relative to subjects 3–6 months old during sleep (*p* < 0.05, Figure 2B).

### 3.2 wPLI FCNs in the Alpha Band Increase in Strength With Age, Particularly in the Frontocentral Region

**FIGURE 3:**
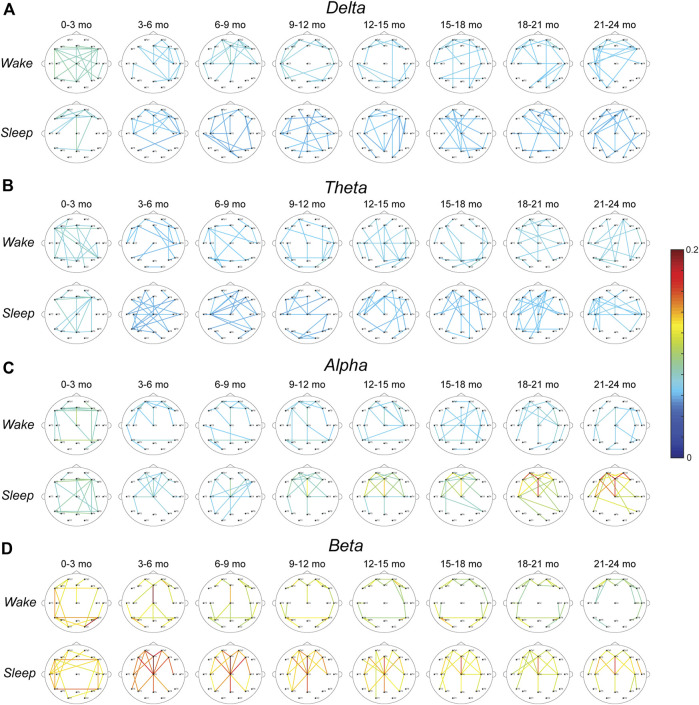
Average wPLI FCNs for healthy infants in the **(A)** delta, **(B)** theta, **(C)** alpha, and **(D)** beta band during wakefulness (top) and sleep (bottom). The strongest 10% of connections in each age group are shown. The color of each connection represents the connection strength, defined as the percentage of epochs with significant connectivity values.

**FIGURE 4:**
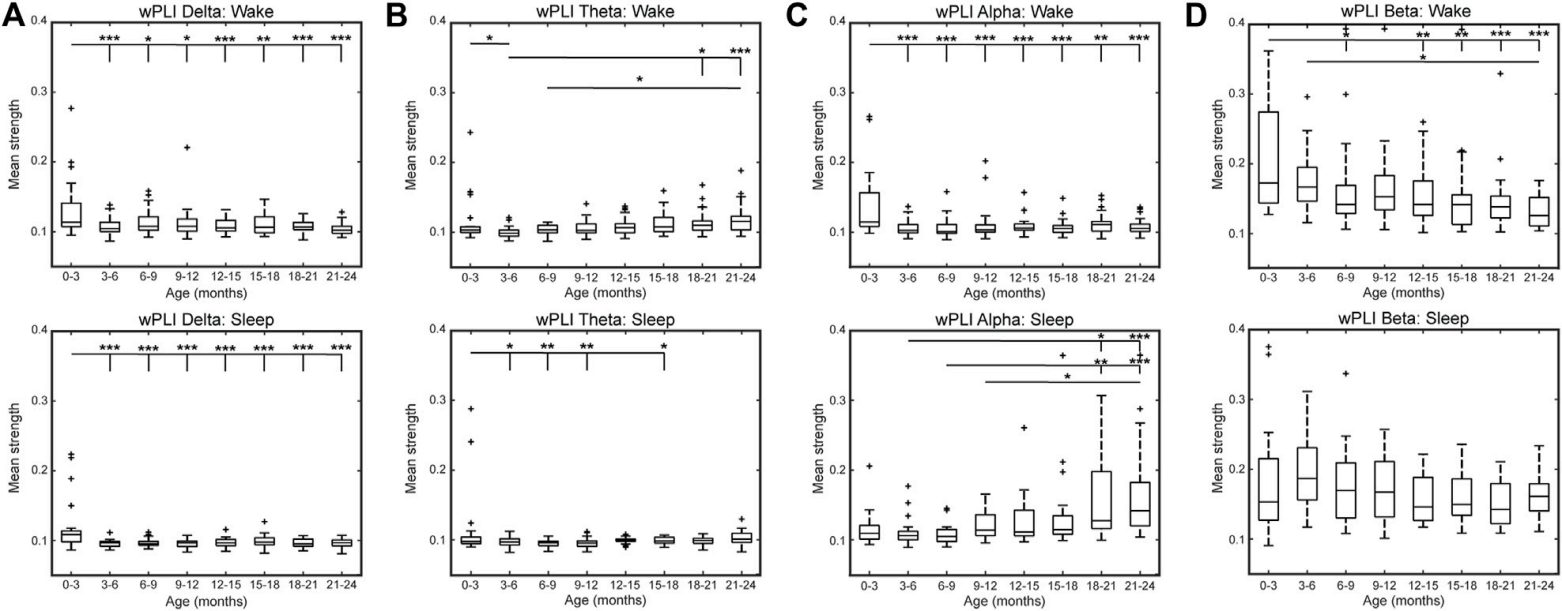
Mean network strength by age using wPLI connectivity in the **(A)** delta, **(B)** theta, **(C)** alpha, and **(D)** beta band during wakefulness (top) and sleep (bottom). Here, mean network strength is defined as the mean of the 10% highest connectivity values. Significance levels are *p < 0.05, **p < 0.01, and ***p < 0.001, with *p*-values modified using the Bonferroni method.

The top 10% strongest wPLI connections for all age groups are shown in Figure 3A (delta band), Figure 3B (theta band), Figure 3C (alpha band), and Figure 3D (beta band). In the delta frequency band, the 0–3-months age group had significantly stronger connectivity than all other age groups, during both wakefulness and sleep (*p* < 0.01, Figure 4A). Connectivity strength in the theta band also decreased from the 0–3-months group to the 3–6 months group during wakefulness (*p* < 0.05), and to the 3–12 and 15–18 months groups during sleep (*p* < 0.05, Figure 4B). The only significant increases in connectivity strength in the delta and theta frequency bands were found in the theta band during wakefulness, where the FCNs in the 18–24 months group were stronger than in the 3–6 months group, and the 21–24 months group was stronger that the 6–9 months group (*p* < 0.05, Figure 4B).

In the alpha band during wakefulness, the FCN strength exhibited changes similar to those seen in the delta band, with significantly lower mean connectivity for subjects 3–24 months old compared to subjects 0–3 months old (*p* < 0.05, Figure 4C, top). In contrast, during sleep, the alpha band connectivity strength exhibited a significant increase during the first 2 years of life. Specifically, subjects in the 18–21 months age group had significantly stronger FCNs than subjects 3–9 months old (*p* < 0.05, Figure 4C bottom), and subjects 21–24 months old had significantly stronger FCNs than subjects 3–12 months old (*p* < 0.01, Figure 4C, bottom). Qualitatively, this change appeared to be driven by strong frontocentral connections (between electrodes Fp1, Fp2, Fz, F3, F4, F7, F8, C3, Cz, and C4), as the strongest 10% of connections for subjects 12–24 months old tended to be clustered in this region (Figure 3C, bottom).

The strongest 10% of connections in the beta band during sleep were also clustered in the frontocentral regions for subjects 3–24 months old, based on a qualitative visual analysis (Figure 3D). However, unlike the alpha band, there were no significant differences in connectivity strength between age groups (Figure 4D, bottom). During wakefulness, the beta band FCNs exhibited stronger connections for subjects 0–3 months old compared to most age groups from 12 to 24 months old, similar to the trends in the delta and alpha bands during wakefulness (*p* < 0.05, Figure 4D, top). In the beta band, the 3–6 months group also exhibited stronger connections than the 21–24 months group (*p* < 0.05, Figure 4D, top).

### 3.3 CC FCNs Exhibit Small-World Features Across all Age Groups

**FIGURE 5:**
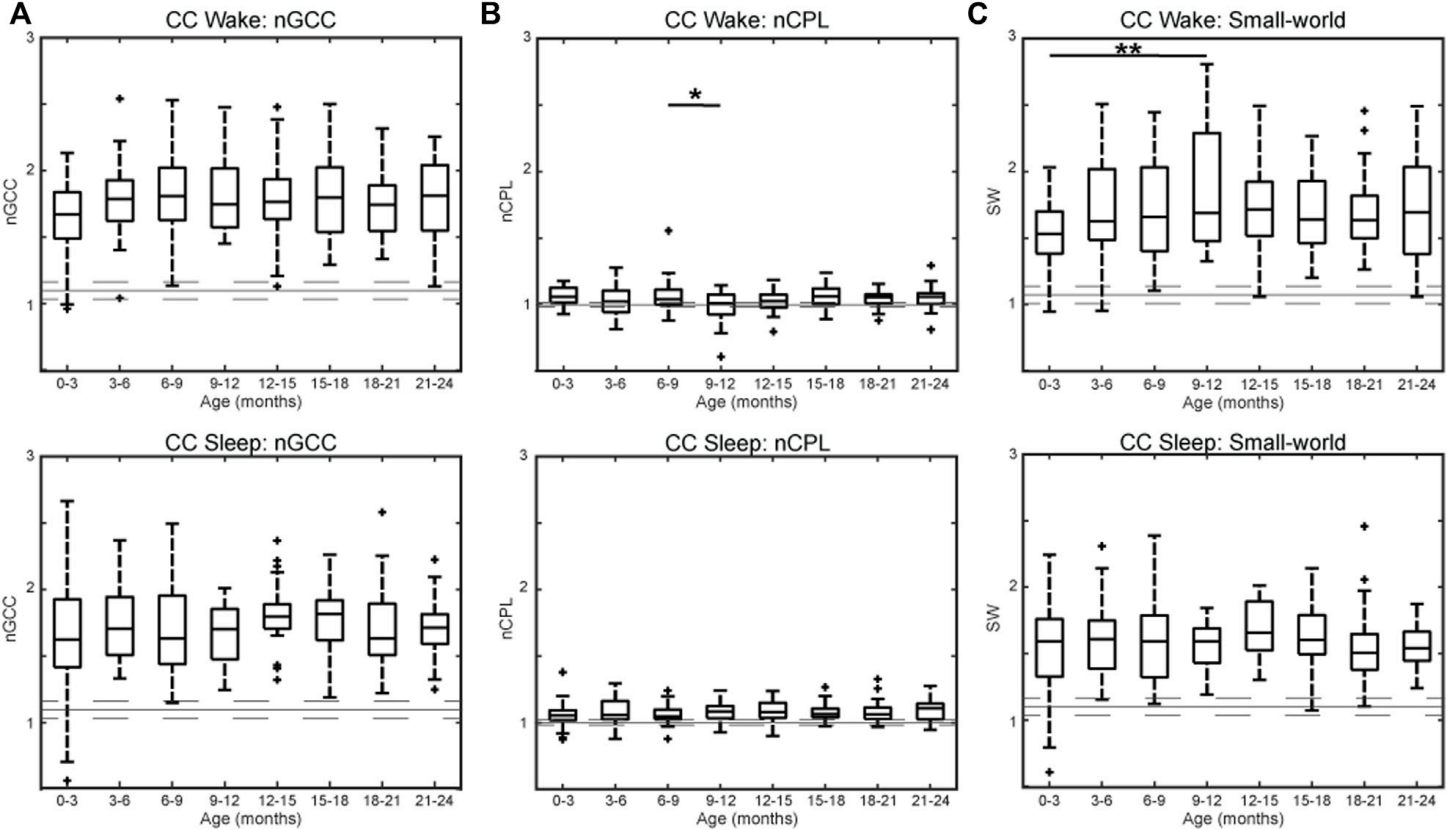
Graph theory measures by age for healthy infants using CC connectivity for the **(A)** normalized global clustering coefficient, **(B)** normalized characteristic path length, and **(C)** small-world measure during wakefulness (top) and sleep (bottom). For each subfigure, the solid gray line represents the median value using randomly rewired networks and the dashed gray lines represent the 25th and 75th percentiles. Significance levels are *p < 0.05, **p < 0.01, and ***p < 0.001, with *p*-values modified using the Bonferroni method.

The nGCC across all subjects indicates the presence of non-random clusters within the network during wakefulness [nGCC_wake_ = 1.75 (0.38), reported as the median (IQR) for all results] and sleep [nGCC_sleep_ = 1.70 (0.39)] (Figure 5A). The nCPL across all subjects was close to one during wakefulness [nCPL_wake_ = 1.05 (0.11)] and sleep [nCPL_sleep_ = 1.06 (0.10)], suggesting that the efficiency of information transfer in the CC network was high, similar to a random network (Figure 5B). CC networks demonstrated a small-world structure across all age groups in wakefulness [SW_wake_ = 1.64 (0.49)] and sleep [SW_sleep_ = 1.59 (0.34)] (Figure 5C). There were no significant differences between age groups for nGCC (wakefulness and sleep), CPL (sleep), or SW (sleep). The nCPL during wakefulness was significantly larger in subjects 6–9 months old compared to 9–12 months old (*p* < 0.05, Figure 5B, top), and SW during wakefulness was significantly larger in subjects 9–12 months old compared to subjects 0–3 months old (*p* < 0.01, Figure 5C, top).

### 3.4 wPLI FCNs Have Less Clustering and are Less Small-World Than CC Networks

**FIGURE 6:**
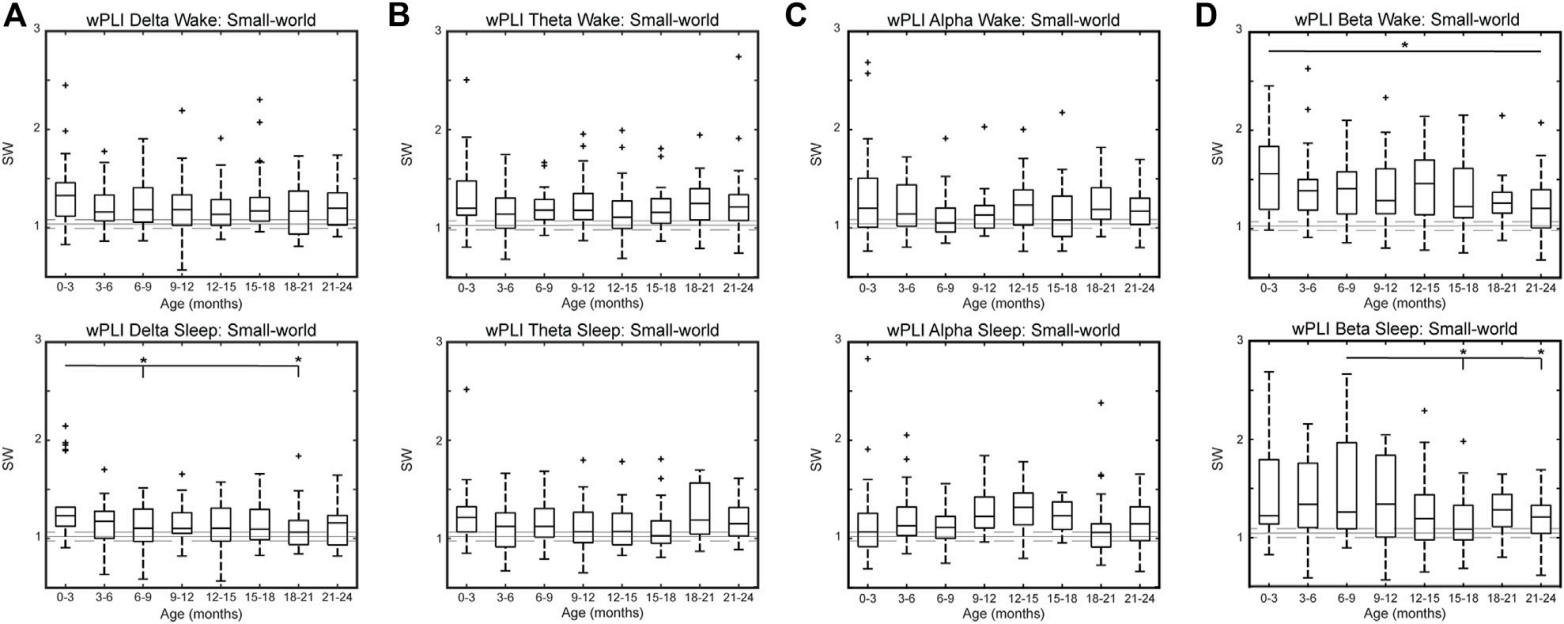
Small-world measure by age for healthy infants using wPLI connectivity in the **(A)** delta, **(B)** theta, **(C)** alpha, and **(D)** beta band during wakefulness (top) and sleep (bottom). For each subfigure, the solid gray line represents the median value using randomly rewired networks and the dashed gray lines represent the 25th and 75th percentiles. Significance levels are *p < 0.05, **p < 0.1, and ***p < 0.001, with *p*-values modified using the Bonferroni method.

WPLI networks generally followed the same trend as CC networks, with nGCC values greater than one and nCPL values of approximately one in all frequency bands. The SW measures for all frequency bands are shown in Figure 6; see Supplementary Figures S6, S7 for the nGCC and nCPL results, respectively. The wPLI FCNs were small-world, with SW values greater than one in the delta [SW_delta, wake_ = 1.18 (0.32), SW_delta, sleep_ = 1.12 (0.28)], theta [SW_theta, wake_ = 1.17 (0.27), SW_theta, sleep_ = 1.12 (0.30)], alpha [SW_alpha, wake_ = 1.15 (0.33), SW_alpha, sleep_ = 1.16 (0.33)], and beta [SW_beta, wake_ = 1.32 (0.46), SW_beta, sleep_ = 1.24 (0.52)] bands. The wPLI FCNs in the delta band had a significantly higher nGCC and SW during sleep at 0–3 months compared to 6–9 months and 18–21 months (*p* < 0.05, Figure 6A bottom and Supplementary Figure S6A bottom). No significant differences in nGCC, nCPL, or SW were seen between age groups in the theta and alpha bands (Figures 6B,C and Supplementary Figures S6B,C, S7B,C). The wPLI networks in the beta band were typically less SW with age, with significantly smaller SW at 21–24 months compared to 0–3 months during wakefulness (*p* < 0.05) and smaller SW at 15–18 and 21–24 months compared to 6–9 months (*p* < 0.05) during sleep (Figure 6D).

The CC networks had significantly higher SW values compared to wPLI networks for all groups aged 3–24 months in the delta, theta, and alpha bands during wakefulness and sleep (Table 2; Wilcoxon sign-rank test, Bonferroni corrected). In the beta band, CC networks were more small-world than wPLI networks across most age groups older than 3 months during wakefulness and 12 months during sleep (Table 2).

**TABLE 2:** *p*-values for the statistical comparisons of the small-world measure between CC and wPLI FCNs in individual subjects. Significant *p*-values are bolded and corrected for FDR using Bonferroni correction (*n* = 8).

State	SW comparison				
	Age group	CC > Delta	CC > Theta	CC > Alpha	CC > Beta
Wake	0–3 m	0.21	0.027	0.063	0.57
	3–6 m	**4.65e** ^**−6**^	**3.10e** ^**−6**^	**6.92e** ^**−6**^	**4.34e** ^**−4**^
	6–9 m	**1.99e** ^**−5**^	**1.35e** ^**−6**^	**1.85e** ^**−6**^	**0.0034**
	9–12 m	**1.24e** ^**−5**^	**4.21e** ^**−6**^	**1.35e** ^**−6**^	**2.12e** ^**−4**^
	12–15 m	**6.92e** ^**−6**^	**6.27e** ^**−6**^	**7.64e** ^**−6**^	**0.0022**
	15–18 m	**8.99e** ^**−5**^	**1.47e** ^**−5**^	**4.26e** ^**−6**^	**5.87e** ^**−4**^
	18–21 m	**2.06e** ^**−6**^	**1.24e** ^**−5**^	**4.65e** ^**−6**^	**3.16e** ^**−5**^
	21–24 m	**9.17e** ^**−5**^	**3.47e** ^**−5**^	**2.40e** ^**−5**^	**1.18e** ^**−4**^
Sleep	0–3 m	0.077	0.012	**0.0010**	0.21
	3–6 m	**2.42e** ^**−5**^	**9.91e** ^**−6**^	**6.96e** ^**−5**^	0.025
	6–9 m	**2.06e** ^**−6**^	**6.92e** ^**−6**^	**1.24e** ^**−5**^	0.17
	9–12 m	**7.66e** ^**−5**^	**1.83e** ^**−5**^	**7.97e** ^**−4**^	0.050
	12–15 m	**3.17e** ^**−5**^	**3.17e** ^**−5**^	**3.17e** ^**−5**^	**2.48e** ^**−4**^
	15–18 m	**6.49e** ^**−6**^	**2.10e** ^**−5**^	**1.00e** ^**−5**^	**2.10e** ^**−5**^
	18–21 m	**1.38e** ^**−4**^	**0.0014**	**5.21e** ^**−4**^	**9.14e** ^**−4**^
	21–24 m	**1.42e** ^**−5**^	**1.42e** ^**−5**^	**4.27e** ^**−5**^	**2.34e** ^**−5**^

Significant *p*-values are bolded and corrected for FDR using Bonferroni Correction(*n* = 8).

## 4 Discussion

In this study, we measured age-related changes in functional connectivity in a large cohort of healthy infants (*n* = 240) using CC and wPLI. CC FCNs maintained a consistent small-world structure (SW > 1), with connections that increased in strength after the first 3 months of life. The wPLI FCNs in the delta and theta bands (wakefulness and sleep), alpha band (wakefulness), and beta band (wakefulness) exhibited significantly decreased strength after 3 months of age. However, in the alpha band during sleep, wPLI connectivity significantly increased in strength in infants older than 21 months. The wPLI networks also exhibited a small world structure, but the locations of the strongest connections differed from the CC networks. For both CC and wPLI, the graph theory metrics showed few significant differences between age groups, suggesting that these features of the network structure stay relatively consistent during infancy.

### 4.1 Connectivity Changes in CC Networks During Infant Development

The CC network structure in the present study was qualitatively similar to that found for healthy infant controls in our prior work, with the strongest connections located in the frontal and posterior head regions (Shrey et al., 2018; Smith et al., 2021). Our prior study also reported stronger FCNs during sleep compared to wakefulness in a cohort of infants with a mean age of 6.3 ± 3.1 months old (Smith et al., 2021), and we saw similar results here in subjects 0–9 months old (Figure 2). Consistent with our results, Gao et al. (2011) found a significant increase in fMRI connectivity strength and global efficiency in the first year of infancy and a stable network strength during the second year. An EEG study using the same CC connectivity method on healthy subjects 0–18 years old during N2 sleep also reported low network strength during infancy that began to significantly increase after 5 years of age (Chu et al., 2014). Our results are generally consistent with prior studies suggesting that FCNs based on broadband EEG strengthen with age, presumably continuing into adulthood. This increase in connectivity strength may also reflect the myelination of white matter tracts during the first year of infancy, which correlates with increased fractional anisotropy on MRI diffusion tensor imaging (Hermoye et al., 2006). While we found significant changes that occur during the first 2 years of development, these changes appear to be subtle relative to those reported later in life.

### 4.2 Connectivity Changes in wPLI Networks During Infant Development

In the delta and theta frequency bands, the 10% strongest connections in the wPLI FCNs appeared highly variable across age groups, likely due to the application of thresholding to relatively weak networks (note the overall low strength for delta and theta in Figure 4). However, wPLI connectivity strength in delta, alpha, and beta frequencies during wakefulness, as well as delta and theta frequencies during sleep, were significantly higher in subjects 0–3 months than other age groups, consistent with prior work describing a decrease in density of FCNs based on EEG coherence during the first 6 months of infancy in the delta, theta, and alpha bands (Chu et al., 2014). The increase in wPLI strength in the alpha band during sleep, with the 10% strongest connections located primarily in frontocentral regions, is complementary to prior wPLI studies of different age groups. For example, stronger alpha and theta band frontoparietal connections were seen in full-term infants relative to preterm newborns, suggesting developmental changes in these frequency bands (Omidvarnia et al., 2014; Tóth et al., 2017). Furthermore, the strongest wPLI functional connections were found in the alpha band in children 5–11 years old (Ortiz et al., 2012; Choi et al., 2019). The increase in alpha band connectivity during N2 sleep at around 21 months may also be related to the fact that sleep spindles are asynchronous in early infancy and become mostly synchronous by age two (Gruber and Wise, 2016; Louis et al., 2016; Goetz et al., 2021). While adult sleep spindles primarily have peak frequencies in the beta band (12–15 Hz), the strong networks we reported in both the alpha and beta bands during N2 sleep are consistent with reports of two types of spindles in children: slow spindles (peak frequency 11–12.75 Hz) occurring in frontal channels (Fz, F3, and F4), and fast spindles (12.5–14.5 Hz) localized to centroparietal channels (Cz and Pz) (Shinomiya et al., 1999). The presence of these two spindle types continues into adulthood, with the peak frequency of both spindle types increasing linearly with age (Shinomiya et al., 1999; Schabus et al., 2007; Mölle et al., 2011).

We found that infant FCNs were small-world across all ages, frequency bands, and states of vigilance, concordant with prior reports of small-world EEG networks immediately following birth (Omidvarnia et al., 2014; Tóth et al., 2017) and during childhood (Boersma et al., 2011). The presence of small-world networks has also been reported in studies utilizing DTI (Yap et al., 2011), volume-based MRI (Fan et al., 2011), and MEG synchronization likelihood (Berchicci et al., 2015), where infant brain networks were small-world at birth and increased in clustering and efficiency with age. We saw a few significant changes in nGCC, nCPL, and SW across different age groups and frequency bands; however, these differences typically involved only two to three age groups and did not suggest a consistent trend related to development. This may be related to the prior suggestion that the most dramatic changes in GT measures occur later in life, from childhood to adulthood (Chu et al., 2014; Berchicci et al., 2015).

### 4.3 Differences Between CC and wPLI Connectivity

We chose to quantify and characterize the functional networks of our subjects using two complementary computational techniques: cross-correlation and weighted phase lag index. Prior work has shown that the differences in networks produced by each method cannot wholly be explained by the differences in sensitivity to linear and nonlinear features of the data, suggesting that the results may be affected by other unknown elements as well (Siems and Siegel, 2020). The differences in FCNs using different connectivity techniques may be explained by the distinct neural mechanisms underlying cortical phase- and amplitude-coupling, which are also frequency specific (Tokariev et al., 2016; Siems and Siegel, 2020). Our results indicated several differences between CC and wPLI FCNs, notably an increase in CC connectivity strength after 3 months of age contrasted with a decrease in wPLI strength in the same time frame. Using different connectivity techniques on the same dataset highlighted distinct aspects of the infant functional networks.

The use of bivariate connectivity methods such as CC and wPLI may result in spurious connections in the FCNs due to the presence of volume conduction. In particular, this can occur when one source drives activity in multiple channels, resulting in false connectivity between all secondary channels (Blinowska and Kaminski, 2013). However, the results in the present study should be minimally impacted by this for three reasons: 1) Our application of CC for functional connectivity includes a step to remove zero-lag connections, which has been shown to counteract volume conduction (Chu-Shore et al., 2012). 2) wPLI inherently reduces the impact of volume conduction by minimizing connections with phase differences at zero and pi (Vinck et al., 2011). 3) If we assume that any remaining effects of volume conduction would impact each subject approximately equally, then any differences we noted in FCNs between subject groups (calculated with the same connectivity technique) should not be spuriously arising from volume conducted sources. Future studies could utilize multivariate connectivity methods to minimize any such spurious connections (Blinowska, 2011).

### 4.4 Normalization and Thresholding in FCNs

While our report of a small-world network configuration is consistent with prior literature, the result of SW > 1 is heavily influenced by the choice of thresholding technique applied to the adjacency matrix. Dividing each FCN by its strongest connection addresses the influence of connectivity strength on the nGCC, nCPL, and SW measures, but it may introduce bias to the GT measures because it gives equal weight to all FCNs, even those with very weak connections (van Wijk et al., 2010). Similarly, using a fixed edge density for GCC, CPL, and SW measures can potentially exclude strong edges or include weak edges in the network, causing spurious results (van den Heuvel et al., 2017). Here, we chose to preserve the weight of each connection to create a pseudo-binary network, as a means of retaining the relative strength in each connection and reducing the effect of weaker edges on the GT measures.

### 4.5 Limitations

Our results are limited by several factors that should be addressed in future investigations. The use of clinical infant EEG limits the number of nodes in the FCN to nineteen (corresponding to the number of electrodes), which restricts the topological characterization of the FCNs; future studies may wish to introduce higher density EEGs for more detailed topological analysis. In addition, GT metrics cannot be used to draw conclusion about the specific network structure; for example, dramatically different networks can have similar values for clustering or path length. This could be addressed by applying statistical tests to individual network connections across age groups. It is also worth noting that the EEGs obtained for the study were recorded from infants referred for diagnostic evaluation of suspected seizures. While they were found to be neurologically normal with no abnormal EEG findings, future studies should consider prospective collection of data from healthy infants. Moreover, we did not directly identify and correct for eye movements in the EEG data, which could influence the connectivity results in the frontal brain regions. However, we expect minimal eye movements during N2 sleep, and the most relevant prior literature did not include this pre-processing step (Chu et al., 2014; Shrey et al., 2018; Smith et al., 2021), so the methods used here facilitate the most direct comparison of results. Finally, the use of a cross-sectional population made it impossible to assess developmental changes in individual cases; future developmental studies should aim to include longitudinally collected EEG data.

## 5 Conclusion

Studies of healthy brain networks in the infant brain are critical for understanding both normal brain development and disease states, such as epilepsy. This has the potential to lead to identification of novel functional connectivity biomarkers to aid clinical diagnosis and treatment, improving the care of children with neurological diseases.

## Data Availability

The raw data supporting the conclusion of this article will be made available by the authors, without undue reservation.
