# Supplementary material for: Evolution of Cortical Functional Networks in Healthy Infants
Source: Front Netw Physiol. 2022 Jun 15;2:893826. doi: 10.3389/fnetp.2022.893826 (PMC10013075; doi:10.3389/fnetp.2022.893826)
Supplement: Supplementary file 1 [file DataSheet1.docx]

Supplementary Material

# Supplementary Figures

## Supplementary Figure 1


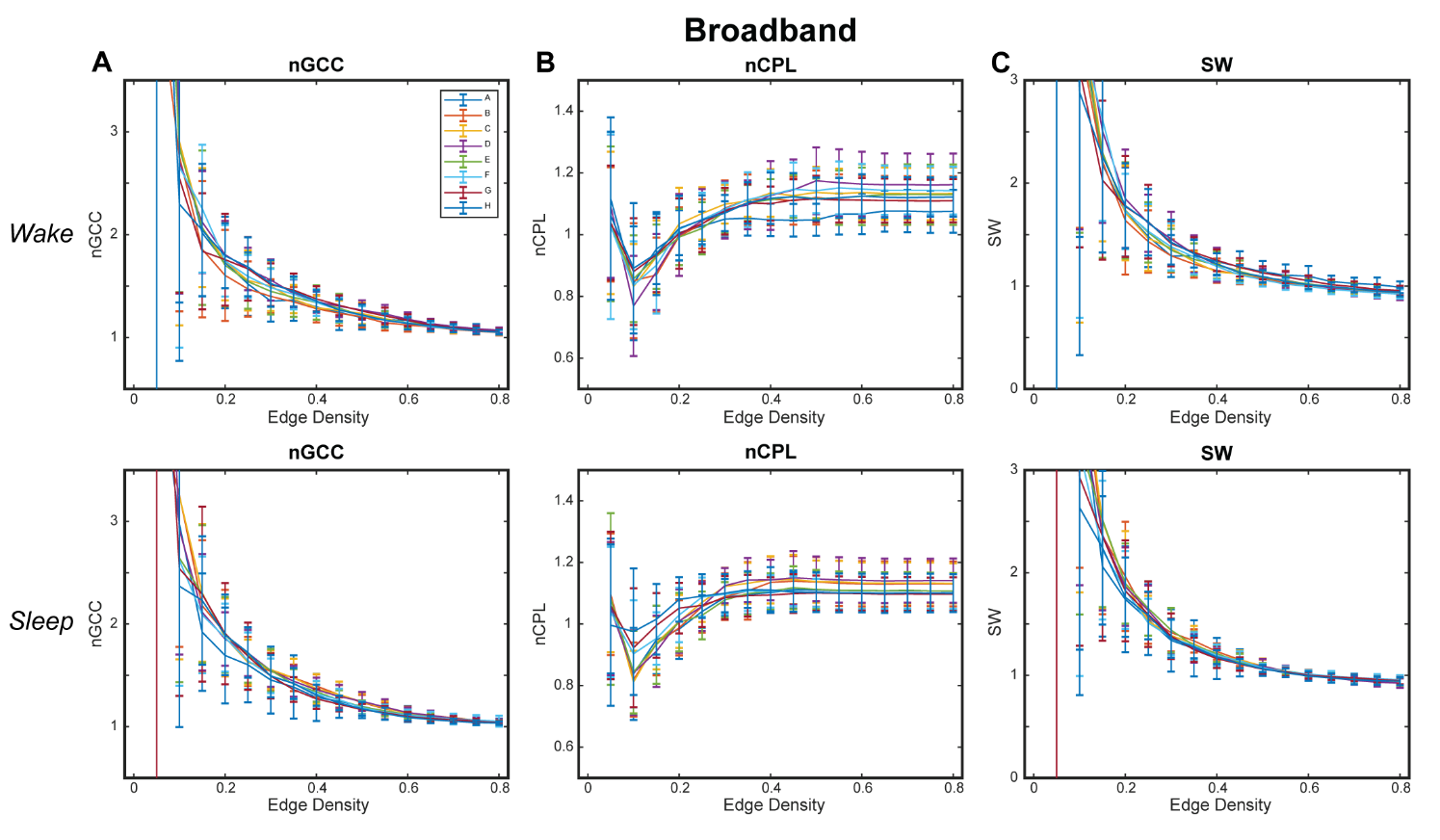


Supplementary Figure 1. Changes in the (A) nGCC, (B) nCPL, and (C) SW as a function of varying edge density thresholds using broadband CC connectivity during wakefulness (top) and sleep (bottom). Edge densities under 10% have high variability and can form unconnected graphs while edge densities over 40% result in networks resembling fully connected graphs, resulting in nGCC and SW values near 1. An edge density of 25% was chosen in the present study due to the lower variability between subjects while forming a connected graph. The overlap in error bars suggest that there is no significant difference between age groups, regardless of the edge density.

## Supplementary Figure 2


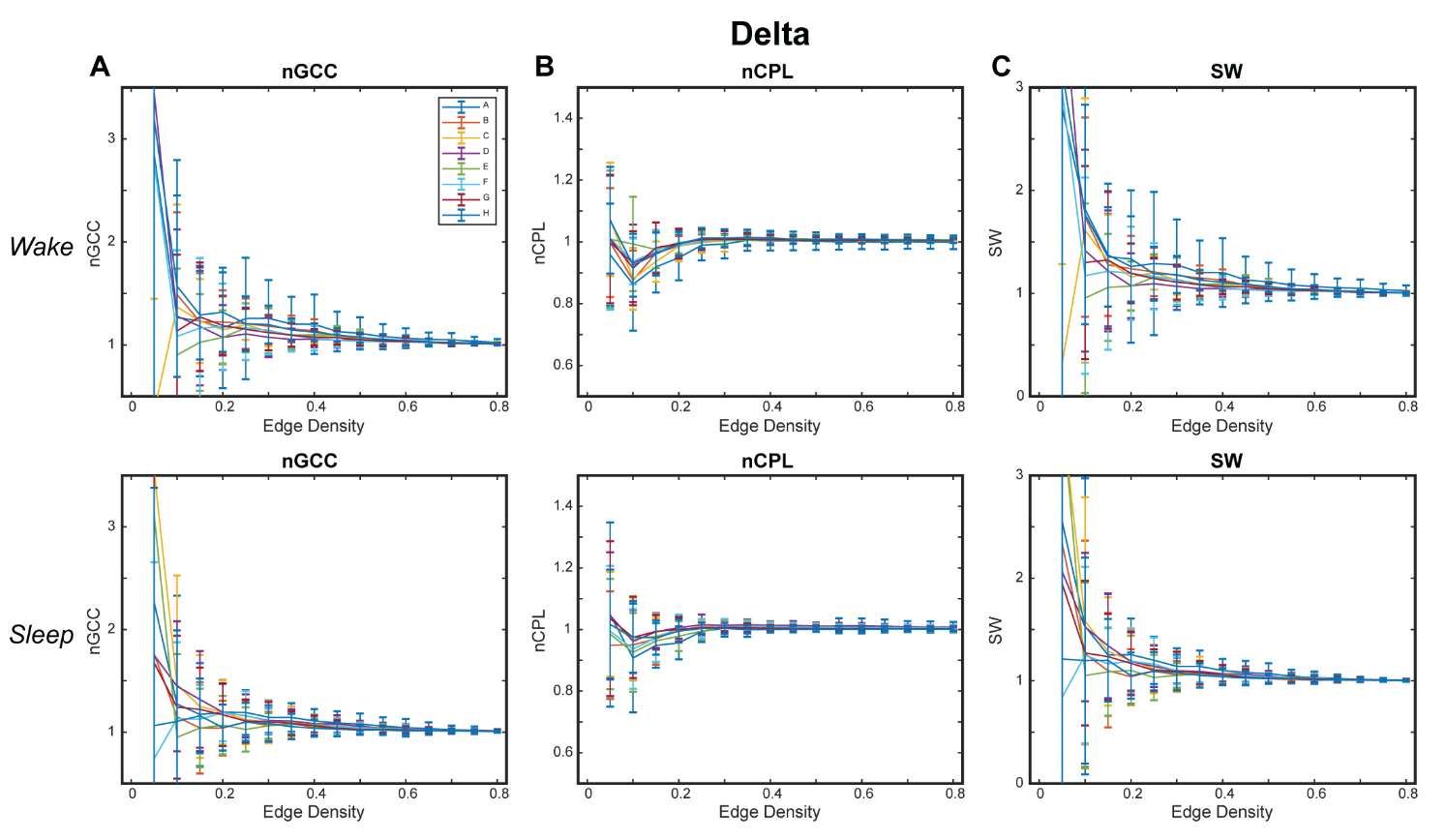


Supplementary Figure 2. Changes in the (A) nGCC, (B) nCPL, and (C) SW as a function of varying edge density thresholds using delta wPLI connectivity during wakefulness (top) and sleep (bottom). Edge densities under 10% have high variability and can form unconnected graphs while edge densities over 40% result in networks resembling fully connected graphs, resulting in nGCC and SW values near 1. An edge density of 25% was chosen in the present study due to the lower variability between subjects while forming a connected graph. The overlap in error bars suggest that there is no significant difference between age groups, regardless of the edge density.

## Supplementary Figure 3


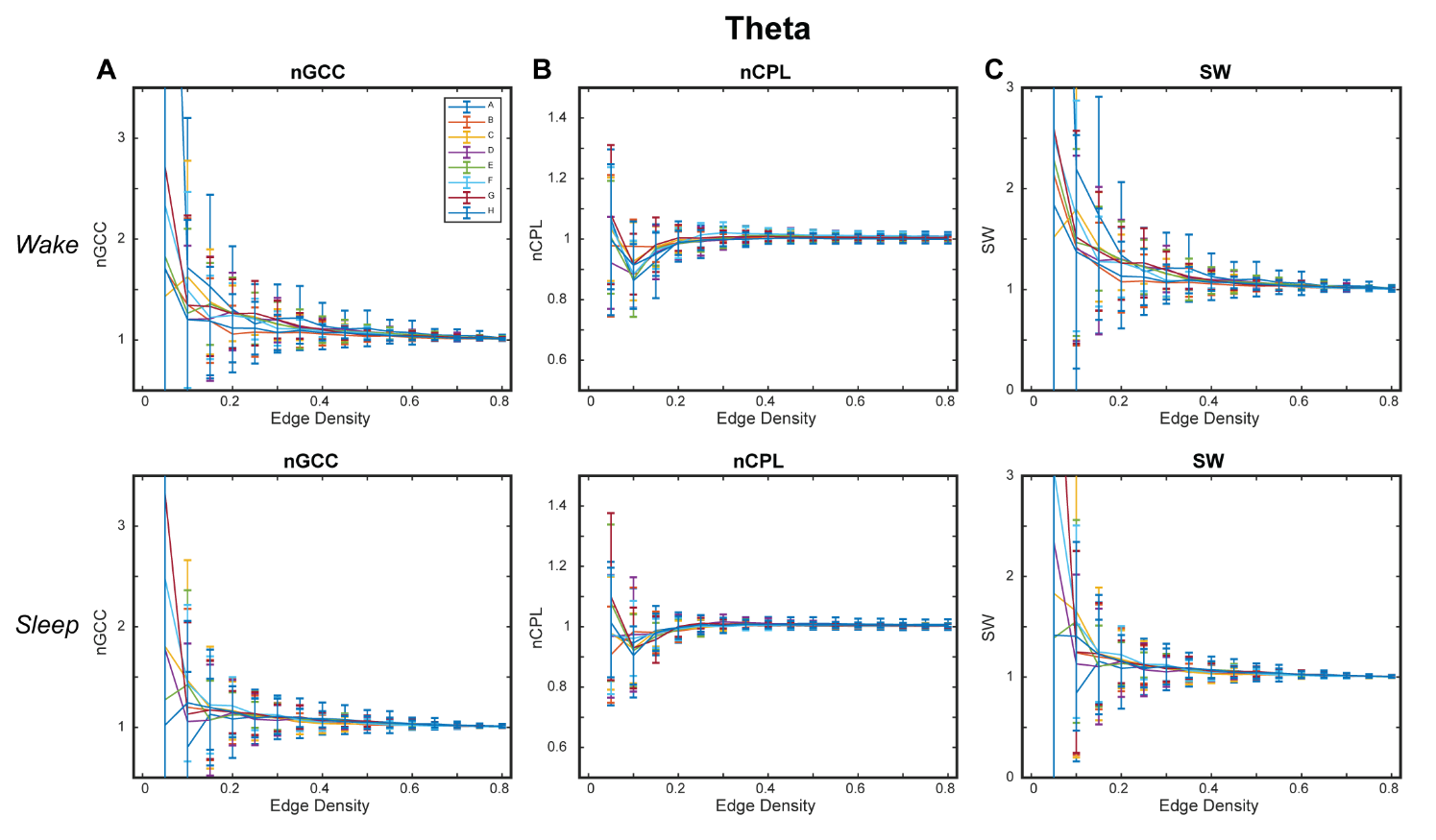


Supplementary Figure 3. Changes in the (A) nGCC, (B) nCPL, and (C) SW as a function of varying edge density thresholds using theta wPLI connectivity during wakefulness (top) and sleep (bottom). Edge densities under 10% have high variability and can form unconnected graphs while edge densities over 40% result in networks resembling fully connected graphs, resulting in nGCC and SW values near 1. An edge density of 25% was chosen in the present study due to the lower variability between subjects while forming a connected graph. The overlap in error bars suggest that there is no significant difference between age groups, regardless of the edge density.

## Supplementary Figure 4


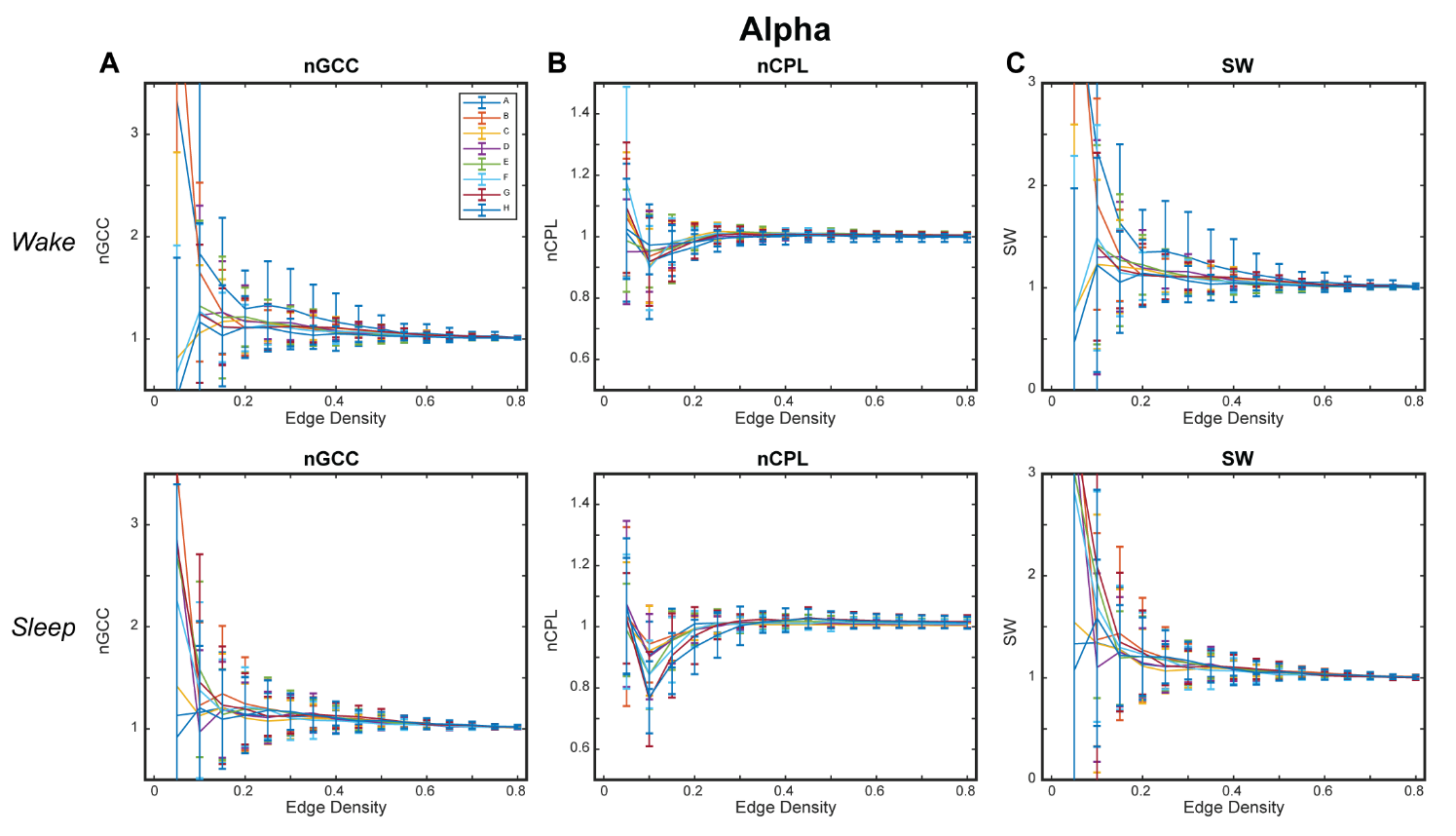


Supplementary Figure 4. Changes in the (A) nGCC, (B) nCPL, and (C) SW as a function of varying edge density thresholds using alpha wPLI connectivity during wakefulness (top) and sleep (bottom). Edge densities under 10% have high variability and can form unconnected graphs while edge densities over 40% result in networks resembling fully connected graphs, resulting in nGCC and SW values near 1. An edge density of 25% was chosen in the present study due to the lower variability between subjects while forming a connected graph. The overlap in error bars suggest that there is no significant difference between age groups, regardless of the edge density.

## Supplementary Figure 5


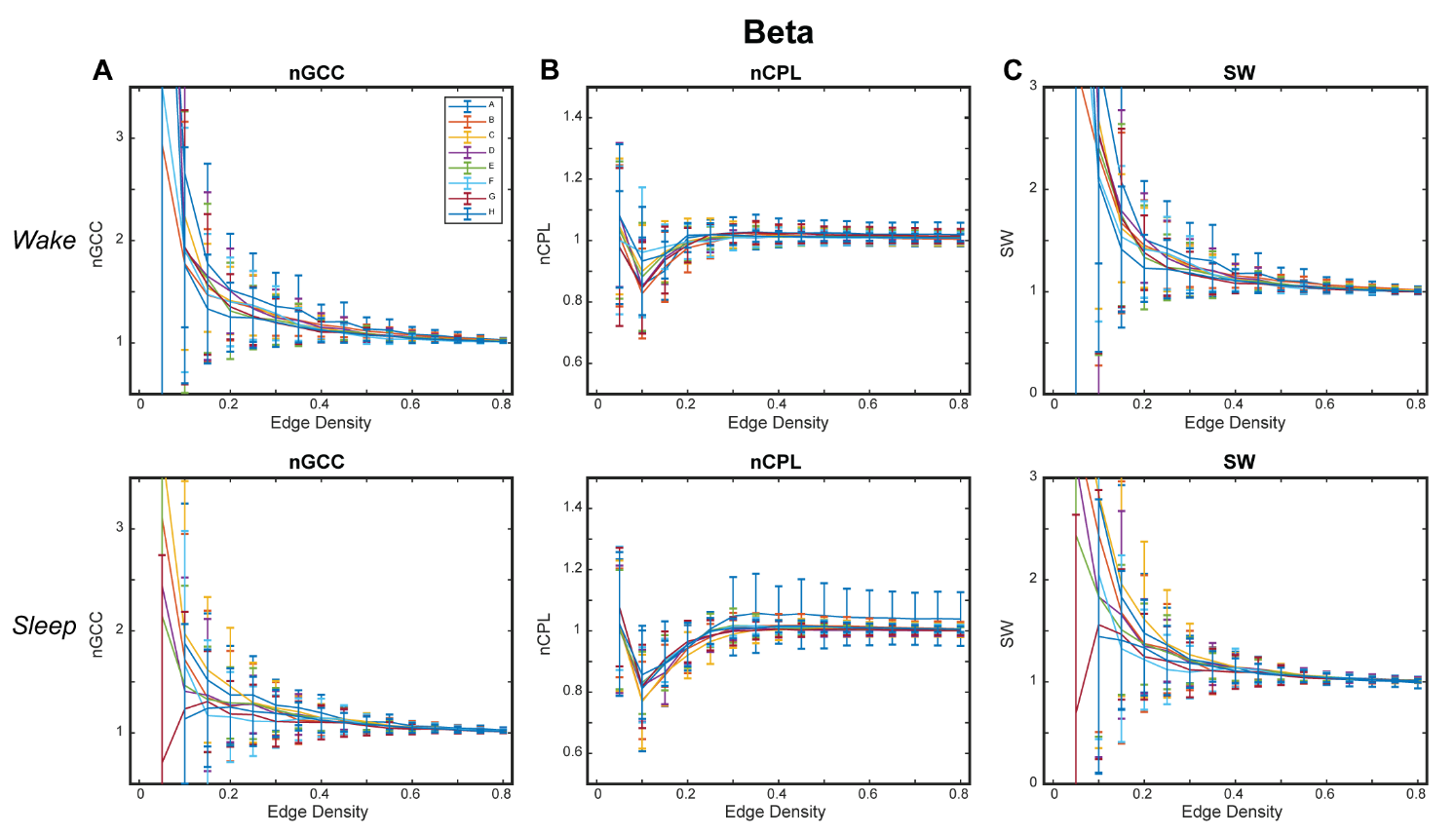


Supplementary Figure 5. Changes in the (A) nGCC, (B) nCPL, and (C) SW as a function of varying edge density thresholds using beta wPLI connectivity during wakefulness (top) and sleep (bottom). Edge densities under 10% have high variability and can form unconnected graphs while edge densities over 40% result in networks resembling fully connected graphs, resulting in nGCC and SW values near 1. An edge density of 25% was chosen in the present study due to the lower variability between subjects while forming a connected graph. The overlap in error bars suggest that there is no significant difference between age groups, regardless of the edge density.

## Supplementary Figure 6


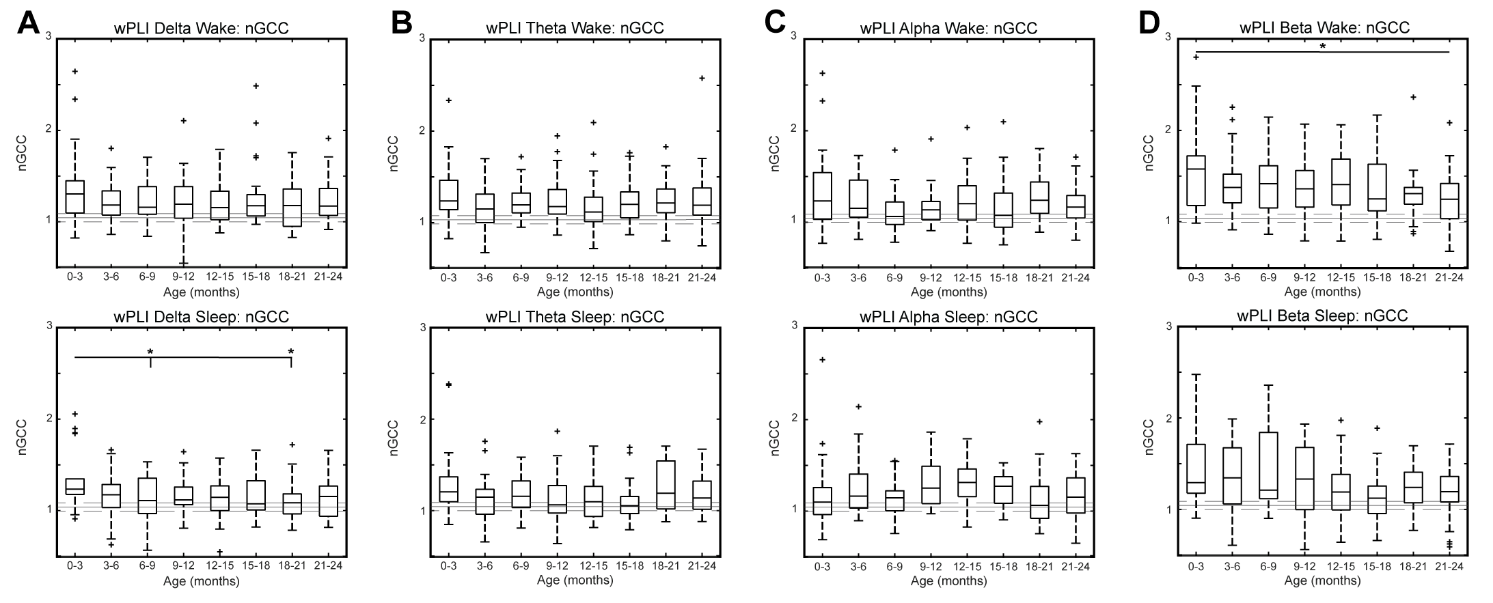


Supplementary Figure 6. Normalized global clustering coefficient (nGCC) by age for healthy infants using wPLI connectivity in the (A) delta, (B) theta, (C) alpha, and (D) beta bands during wakefulness (top) and sleep (bottom). The solid line represents the median values using randomly rewired networks and the dashed lines represent the 25^th^ and 75^th^ percentiles. Significance levels are * p<.05, ** p<.01, *** p<.001, with p-values modified using the Bonferroni method.

## Supplementary Figure 7


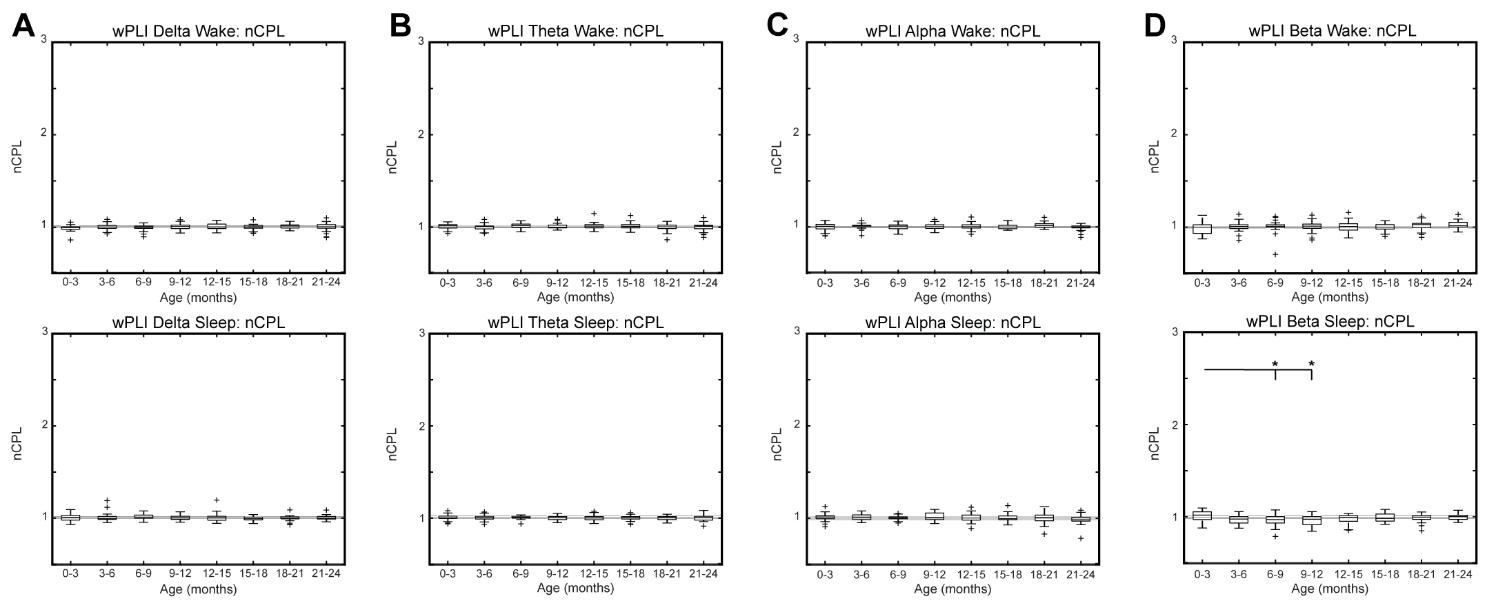


Supplementary Figure 7. Normalized characteristic path length (nCPL) by age for healthy infants using wPLI connectivity in the (A) delta, (B) theta, (C) alpha, and (D) beta bands during wakefulness (top) and sleep (bottom). The solid line represents the median values using randomly rewired networks and the dashed lines represent the 25^th^ and 75^th^ percentiles. Significance levels are * p<.05, ** p<.01, *** p<.001, with p-values modified using the Bonferroni method.

# Supplementary Tables

## Supplementary Table 1

*Mean EEG, artifact, wake, and sleep duration for each age group. The mean EEG duration also includes N1 and N3 sleep*

| **Group** | **Mean EEG duration-seconds (std), [range]** | **Mean artifact duration-seconds [std, range]** | **Mean wakefulness duration-seconds [std, range]** | **Mean quiet/N2 sleep duration-seconds [std, range]** |
| --- | --- | --- | --- | --- |
| *0-3 m.* | 2530 (814)  [1740-3900] | 1105 (500)  [413-2291] | 480 (314)  [109-1255] | 562 (425)  [0-1557] |
| *3-6 m.* | 2110 (355)  [1560-3060] | 485 (204)  [92-969] | 780 (429)  [202-1757] | 534 (312)  [0-1183] |
| *6-9 m.* | 2136 (384)  [1320-3060] | 396 (134)  [227-877] | 689 (389)  [211-1606] | 739 (303)  [143-1525] |
| *9-12 m.* | 2204 (459)  [1200-3360] | 502 (207)  [224-1027] | 877 (448)  [0-1928] | 597 (355)  [0-1178] |
| *12-15 m.* | 2194 (291)  [1807-2940] | 501 (168)  [228-927] | 844 (425)  [212-1853] | 692 (440)  [0-1451] |
| *15-18 m.* | 2318 (370)  [1800-3120] | 566 (309)  [266-1980] | 873 (510)  [123-2051] | 614 (309)  [0-1410] |
| *18-21 m.* | 2254 (397)  [1800-3420] | 532 (234)  [281-1474] | 948 (531)  [188-2024] | 522 (355)  [0-951] |
| *21-24 m.* | 2288 (342)  [1800-3420] | 521 (192)  [258-1114] | 928 (497)  [199-2002] | 570 (343)  [0-1236] |

## Supplementary Table 2

*Mean percentage of EEG artifact for each age group, calculated across the entire EEG recording. The mean percentage of artifact across all subjects was 25%.*

| **Group** | **Artifact % (std)** |
| --- | --- |
| *0-3 m.* | 43.5 (12.9) |
| *3-6 m.* | 22.8 (7.7) |
| *6-9 m.* | 18.9 (6.2) |
| *9-12 m.* | 23.0 (8.4) |
| *12-15 m.* | 22.9 (7.3) |
| *15-18 m.* | 25.0 (15.9) |
| *18-21 m.* | 23.4 (7.6) |
| *21-24 m.* | 22.6 (6.8) |
